# Supplementary material for: Size control and oscillations of active droplets in synthetic cells
Source: Nat Commun. 2025 Feb 26;16:2003. doi: 10.1038/s41467-025-57240-8 (PMC11865504; doi:10.1038/s41467-025-57240-8)
Supplement: Supplementary file 2 — Description of Additional Supplementary Files [file 41467_2025_57240_MOESM2_ESM.pdf]

Description of Additional Supplementary Files

Title: Supplementary Movie 1
Description: **Oscillations in the spatial distribution of synthetic organelles within a synthetic cell with 3.3 mM fuel.**
XY and XZ projections timelapses of a synthetic cell of radius 24.5 μm prepared from confocal Z-stacks. The synthetic cell contains 10 mM peptide, 5 mM PSS polyanion, and 200 mM MES buffer and is embedded in oil with 250 mM DIC as fuel. This fuel is continuously exchanged at the droplet-oil interface. The distribution of the artificial organelles oscillates with time.

Title: Supplementary Movie 2
Description: **Active droplets in steady-state shrink between fusion events.**
Timelapse video of a Z-projection from a Z-stack acquired via confocal microscopy. The sample consists of 14 mM peptide, 15 mM PSS, and 0.2 μM sulforhodamine B in 200 mM MES buffer (pH 5.3), covered by a layer of DIC. The timelapse captures the dynamic behavior of droplets, which shrink between fusion events in the absence of a gel matrix.

Title: Supplementary Movie 3
Description: **Size evolution of droplets nucleated within a gel matrix.**
Z-projection timelapse acquired via confocal microscopy. 14 mM peptide, 15 mM PSS, and 0.2 μM sulforhodamine B were dissolved in 200 mM MES buffer (pH 5.3) with 0.5 w/v% of agarose. The sample was covered by a layer of DIC to supply a constant influx of fuel. Droplets evolve to similar sizes independently of the nucleation time and remain stable after reaching the stable size.

Title: Supplementary Movie 4
Description: **Size evolution of prenucleated droplets within a gel matrix.**
Z-projection timelapse acquired via confocal microscopy. 14 mM peptide, 15 mM PSS, 20 mM EDC and 0.2 μM sulforhodamine B were dissolved in 200 mM MES buffer (pH 5.3) with 0.5 w/v% of agarose, covered by a layer of DIC. In this case, droplets fused before gelification yielding a wide size distribution. Droplets correct their size to a narrow size distribution.

Title: Supplementary Movie 5
Description: **Oscillations in the spatial distribution of synthetic organelles in a synthetic cell at 0.9 mM fuel.**
XY and XZ projections timelapses of a synthetic cell of radius 25.6 μm prepared from confocal Z-stacks. The synthetic cell contains 10 mM peptide, 5 mM PSS polyanion, and 200 mM MES buffer and is embedded in oil with 70 mM DIC as fuel. This fuel is continuously

exchanged at the droplet-oil interface. The distribution, number and size of the artificial organelles oscillates with time.
